# High-Dose Methotrexate at All Ages: Safety, Efficacy, and Outcomes from the HDMTX European Registry

**DOI:** 10.3390/cancers18010124

**Published:** 2025-12-30

**Authors:** Bertrand Pourroy, Maria D. Aumente, Christian Koenecke, Martin Stanulla, Andrés J. M. Ferreri, Thais Murciano-Carillo, Madhumita Dandapani, Timothy A. Ritzmann, Pere Barba, Etienne Chatelut, Katrina M. Ingley, Emma Morris, Elisabeth Schorb, Sven Liebig, Stefan Schwartz, Scott C. Howard, Ryan Combs, Nicolás Tentoni, Jennifer Lowe, Gabriela Villanueva, Claudia Sampor, Miriam Hwang, Carmelo Rizzari

**Affiliations:** 1 Oncopharma Unit, Hôpitaux Universitaires de Marseille La Timone, 13005 Marseille, France; 2 Pharmacy Service, Reina Sofia University Hospital, 14004 Cordoba, Spain; 3 Department of Pediatric Hematology and Oncology, University of Hannover, 30167 Hannover, Germany; 4 Lymphoma Unit, IRCCS San Raffaele Scientific Institute, 20132 Milano, Italy; 5 Pediatric Oncology & Hematology, Hospital Universitari Vall d’Hebron, 08035 Barcelona, Spain; 6 Department of Pediatrics Oncology/Haematology, University of Nottingham, Nottingham NG5 1PB, UK; 7 Children’s Brain Tumour Research Centre, University of Nottingham, Nottingham NG7 2UH, UK; 8 Hematology Department, Hospital Universitari Vall d’Hebron, 08035 Barcelona, Spain; 9 Department of Pharmacology, Institut Universitaire du Cancer-Oncopole, 31059 Toulouse, France; 10 Sarcoma Department, University College London Hospital, London NW1 2PB, UK; 11 Pharmacy Cancer Services, University College London Hospital, London NW1 2PB, UK; 12 Comprehensive Cancer Center, University of Freiburg, 79106 Freiburg, Germany; 13 Department of Hematology, Oncology and Tumor Immunology, Charite Universitätsmedizin, 10117 Berlin, Germany; 14 Resonance, Memphis, TN 38104, USA; 15 Global Development Office, Hospital Sant Joan de Deu, 08950 Barcelona, Spain; 16 Department of Pediatrics, University of Milano-Bicocca, 20126 Milan, Italy

**Keywords:** high-dose methotrexate, delayed methotrexate elimination, acute kidney injury, clinical outcomes

## Abstract

**Objectives**: To determine the incidence of delayed methotrexate elimination (DME) and acute kidney injury (AKI) and their associations with clinical outcomes in patients receiving high-dose methotrexate (HDMTX) for cancer treatment. **Methods**: The HDMTX European Registry collected medical records data from 12 institutions in 5 European countries to investigate the clinical practice patterns of healthcare providers utilizing HDMTX for cancer treatment. Cancer types included were acute lymphoblastic leukemia (ALL), primary central nervous system lymphoma (PCNSL), non-Hodgkin lymphoma (NHL), osteosarcoma, and other CNS cancers. Primary endpoints were the incidence of DME and AKI; secondary endpoints were clinical outcomes, including hospital length of stay (LOS), delay in the subsequent course of treatment, methotrexate dose reduction, and omission of next course of treatment. Associations between the primary and secondary endpoints were analyzed with Chi-square and Wilcoxon rank-sum tests. **Results**: Among the 2501 total HDMTX courses analyzed, DME occurred in 302 courses (12.1%), and AKI in 384 courses (15.4%). DME incidence was highest in courses for PCNSL (18.2%) and NHL (17.2%); AKI incidence was highest in ALL courses (21.0%). Incidence of DME and AKI varied by age and methotrexate infusion duration among the different cancer types. Occurrence of DME was associated with longer delays prior to the next course of treatment, longer hospital LOS, and more frequent methotrexate dose reductions and dose omissions. **Conclusions**: While HDMTX is a very effective and safe treatment, administration of efficacious doses of methotrexate can lead to AKI and DME, and no single or combination of patient or treatment factors was found to reliably predict their occurrence. Thus, diligent monitoring of methotrexate levels is imperative for early detection and prompt management of nephrotoxicity in all settings where HDMTX treatment is administered.

## Simple Summary

This paper describes the incidence of delayed methotrexate elimination and acute kidney injury related to high-dose methotrexate (HDMTX) treatment in various cancers and reports their association with clinical outcomes from 12 treatment centers in 5 European countries. These real-world outcomes outside of the clinical trial setting provide insight into important aspects of HDMTX treatment that should be considered to provide safe and efficacious treatment.

## 1. Introduction

High-dose methotrexate (HDMTX) is an integral component of treatment for various cancers. The potential for significant nephrotoxicity and delayed renal elimination of methotrexate, however, can lead to severe toxicities in other organ systems and premature treatment cessation [1]. The incidence of HDMTX-associated acute kidney injury (AKI) is reported to range between 2% and 30% depending on patient age, dose of methotrexate, and duration of infusion, but may occur without any identifiable factors [2,3,4,5,6,7]. As AKI is usually asymptomatic (non-oliguric), a high level of awareness is warranted in assessing renal function by monitoring serum creatinine concentration (Scr) prior to, during, and after completion of HDMTX infusion. While an increase in Scr of 50% or greater from baseline is established as an indicator of AKI, the lag in elevation of creatinine levels following the actual onset of AKI renders it a suboptimal marker [8,9,10]. Thus, if available, serial monitoring of plasma methotrexate concentration (MTXc) following the start of HDMTX infusion allows detection of delayed methotrexate elimination (DME) and facilitates early intervention to mitigate its toxicity. The definition of DME varies by study, but MTXc ≥ 1 μM at 42 to 48 h or >0.2 μM at 72 h after the end of a 24 h HDMTX infusion or MTXc ≥ 10 μM at 24 h following a short infusion typically indicates the presence of DME [11,12,13]. To encompass all lengths of HDMTX infusion duration, methotrexate levels that are significantly elevated greater than 2 standard deviations of the measured methotrexate excretion curve can be used as a criterion for DME [14].

It is imperative that supportive measures (e.g., hyperhydration, urine alkalinization, leucovorin rescue) are carried out in conjunction with HDMTX treatment to prevent precipitation of methotrexate crystals in the renal tubules and minimize methotrexate cytotoxicity in non-cancer cells [11,12,15]. In cases of DME, prompt administration of glucarpidase within 48–60 h of the start of infusion can prevent the development of irreversible toxicities and increase the odds of renal recovery [3,16,17]. Glucarpidase plays an important role in the management of DME caused by renal impairment as it rapidly metabolizes over 95% of plasma methotrexate within 15 min of intravenous administration and maintains its effect for 48 h [18,19,20].

The High-Dose Methotrexate European Registry (hereafter referred to as “Registry”) was established in 2022 to systematically investigate the clinical practice patterns of healthcare providers utilizing HDMTX to treat various cancers (ClinicalTrials.gov identifier: NCT05899751). Data were collected from 12 institutions in 5 European countries in which HDMTX treatment was provided to cancer patients diagnosed between January 2010 and June 2021. The Registry aimed to investigate characteristics of HDMTX treatment (dose, infusion duration, number of courses) by cancer type, determine the incidence of HDMTX-associated AKI and DME and their impact on treatment outcomes, and document supportive care measures including hyperhydration, urine alkalinization, leucovorin rescue, and glucarpidase administration. This paper reports pertinent findings from the Registry to provide real-world data on all aspects of HDMTX treatment and outcomes during a recent 12-year period. Specifically, the objectives of this study were to (1) quantify the incidence of HDMTX-associated AKI and DME among children and adults diagnosed with acute lymphoblastic leukemia (ALL), primary central nervous system lymphoma (PCNSL), non-Hodgkin lymphomas (NHL), osteosarcoma, and other CNS cancers, and (2) determine the association between occurrence of AKI and DME with treatment characteristics (e.g., methotrexate dose and infusion duration, concomitant medications) and outcomes (e.g., hospital length of stay, methotrexate dose reduction, treatment cessation, adverse events, long-term survival).

## 2. Methods

This was an international multicenter retrospective cohort study to analyze real-world registry data collected from investigational sites selected to achieve a balance between pediatric and adult populations.

### 2.1. Setting

Data were extracted from medical records at 12 sites in 5 European countries: 3 from Spain, 3 from Germany, 2 from Italy, 2 from France, and 2 from the United Kingdom. Data were entered for consecutively diagnosed patients starting with the most recent patients (those diagnosed in 2021) and working backward by year to avoid any bias in patient accrual by assuring that all patients treated during a given year were included without bias.

### 2.2. Participants

Patients were enrolled in the Registry if (1) a cancer was diagnosed from 1 January 2010 to 30 June 2021; (2) HDMTX chemotherapy (methotrexate dose ≥ 500 mg/m^2^ of body surface area [BSA] infused over 1–36 h) was administered; and (3) they had at least one course containing MTXc and Scr measurements following HDMTX infusion. For this analysis, a sub-sample of patients from the Registry was included if data were available for (1) patient age, sex, and BSA at the time of HDMTX administration; (2) methotrexate dose and infusion length; (3) baseline Scr; and (4) at least one post-infusion Scr and MTXc measurement in all their courses.

### 2.3. Endpoints

Primary endpoints were AKI, DME, and DME with concomitant AKI (DME + AKI). AKI was defined as an increase in Scr of greater than 50% of baseline or a nominal increase of 0.3 mg/dL or greater following HDMTX infusion according to the Acute Kidney Injury Network (AKIN) criteria [21]. The AKIN criteria stratifies severity of AKI as follows: Grade 1, an increase in Scr by at least 0.3 mg/dL within 48 h or an increase of 1.5–1.9 times baseline within seven days; Grade 2, Scr increase of 2.0–2.9 times baseline; and Grade 3, Scr increase ≥ 3 times baseline, Scr ≥ 4.0 mg/dL, or the initiation of renal replacement therapy. For this study, severe AKI was defined as meeting the AKIN criteria for Grade 2 or 3 during a course of HDMTX treatment. DME was determined to have occurred if MTXc was greater than 2 standard deviations of the population mean, as simulated by MTXPK.org at 36, 42, or 48 h from the start of HDMTX infusion [22]. Briefly, MTXPK.org is a validated pharmacokinetic modeling tool that simulates MTXc based on available data points that did not meet the criteria for early levels and allows for a standardized and clinically relevant metric for assessing DME [23,24]. Primary endpoints were classified as binary based on whether the event occurred or not during the HDMTX course under consideration.

Secondary endpoints were clinical outcomes including hospital length of stay (LOS), delay to the start of the subsequent block of HDMTX (i.e., 7-days or longer difference between patient-specific protocol prescribed treatment start time and actual start time), methotrexate dose reduction (a decrease of 25% or more from the prior course), methotrexate dose omission in the subsequent course, estimated event-free survival (EFS), and overall survival (OS) from the start of the first course of HDMTX administration.

### 2.4. Covariables

Other variables collected include baseline demographic characteristics, HDMTX administration details (dose, infusion duration), and supportive therapy (e.g., leucovorin rescue, glucarpidase administration). Administration of concomitant medications (e.g., loop diuretics, proton-pump inhibitors) was analyzed to determine their association with the primary endpoints. Abnormal laboratory values (e.g., elevated alanine transaminase [ALT] and bilirubin levels, neutropenia, thrombocytopenia) were recorded and analyzed to determine associations with the primary endpoints.

### 2.5. Statistical Methods

Statistical analyses were performed using R version 4.4.1 (R Foundation for Statistical Computing, Vienna, Austria). A two-sided *p*-value of <0.05 was considered statistically significant for all analyses. Descriptive statistics were used to summarize the demographic characteristics of the patients and relevant clinical information. Continuous variables were presented as median and interquartile range (IQR). Categorical variables were summarized using absolute frequencies and percentages.

Primary endpoint analyses included frequency tables and incidences with 95% exact Clopper–Pearson confidence intervals (95% CIs) for AKI, DME, and DME + AKI. For continuous secondary endpoints (e.g., hospital LOS), median and interquartile range were used to describe their distribution. For nominal dichotomous secondary outcomes (hospital readmission for toxicity management, delay to the start of the subsequent block of HDMTX, and methotrexate dose reduction/omission), incidences with 95% CI were used. For time-to-event secondary endpoints, the Kaplan–Meier estimator was used to assess EFS and OS from 1 to 5 years and compared by the occurrence of AKI, DME, and cancer type using the log-rank test. Chi-square and Wilcoxon rank-sum tests were used to determine associations of the categorical and continuous secondary endpoints, respectively, by the presence of the primary endpoints.

## 3. Results

**Figure 1:**
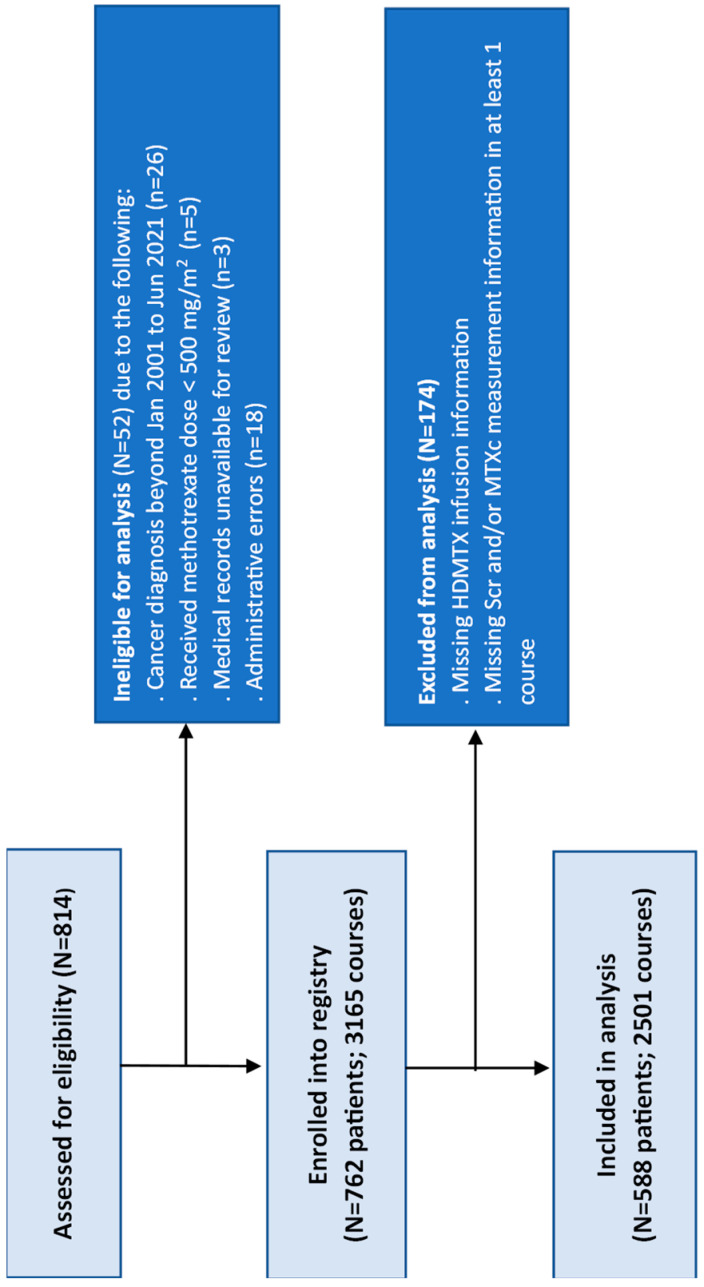
Analysis population.

Among a total of 814 patients assessed for eligibility, 762 patients who received 3165 HDMTX courses were included in the Registry, of which 588 patients with 2501 courses had data that met the criteria for inclusion in analysis (Figure 1).

The distribution of the primary cancers among the 588 patients were 283 with ALL (48.1%), 158 with PCNSL (26.9%), 108 NHL (18.4%), 29 osteosarcoma (4.9%), and 10 (1.7%) with other CNS cancers (medulloblastoma [n = 5], ependymoma [n = 2], pineoblastoma [n = 1], histology unavailable [n = 2]). The median age of patients was 16.4 years (IQR, 5.6–62.2), with 279 (47.4%) adults and 309 (52.6%) children under the age of 18 (Table 1). Methotrexate administration characteristics collected from the 2501 HDMTX courses revealed the median methotrexate dose standard for all courses was 3.4 g/m^2^ (IQR, 2.7–5.0), with doses varying by cancer type (Table 2); patients with osteosarcoma received the highest median dose of 11.8 g/m^2^ (IQR 11.2–12.0), followed by ALL patients with a median dose of 5.0 g/m^2^ (IQR 4.8–5.0). Infusion duration was longest in ALL patients who received a median of 24.0 h and shortest in both NHL and osteosarcoma patient groups, with a median of 4.0 h. Leucovorin rescue was administered in 95.4% of the courses; 99.6% of courses for ALL received leucovorin, while only 87.4% of courses for NHL and 93.7% of PCNSL courses received leucovorin. Glucarpidase was administered in only eight courses, of which three were ALL courses, and five were PCNSL courses. No patients were reported to have died or received extracorporeal treatment (e.g., dialysis) due to methotrexate-related toxicities.

### 3.1. Primary Endpoints

Among the 2501 courses included for analysis, DME occurred in 302 courses (12.1%), AKI in 384 courses (15.4%), severe AKI in 78 courses (3.1%), and DME + AKI in 106 courses (4.2%) (Table 3). Occurrence of DME was highest in courses for PCNSL at 18.2% followed by courses for NHL (17.2%); courses for osteosarcoma were found to have the lowest occurrence of DME. Occurrences of AKI and severe AKI were found to be highest in courses for ALL patients at 21.0% and 5.4%, respectively, followed by courses in NHL patients at 18.9% and 2.6%, respectively. Occurrence of DME was higher in HDMTX courses administered to adults 18 years of age and older in all cancer types compared to courses in children, with the highest rates found in adults with PCNSL at 18.2% (Supplementary Table S1). Adults receiving courses for ALL had a higher occurrence of AKI compared to children with ALL (35.8% vs. 19.2%); however, rates of AKI were similar for adults and children in NHL and osteosarcoma. Occurrences of DME with concomitant AKI were also more frequent for adults compared to children in courses for ALL (11.3% vs. 3.5%) and NHL (8.8% vs. 3.8%).

Courses that administered methotrexate dose ≥ 2.5 to <4 g/m^2^ had the highest occurrence of DME at 20.2%, followed by doses < 2.5 g/m^2^ (15.2%), while AKI was most frequent in courses given doses of ≥4 to <6 g/m^2^ (20.5%), followed by ≥6 to <10 g/m^2^ (14.9%) and ≥2.5 to <4 g/m^2^ (14.2%). Courses given doses greater than 10 g/m^2^ had the least occurrence of both DME and AKI (Table 4). Courses with infusion duration < 5 h had the highest occurrence of DME (21.3%), while AKI was most frequent in courses with infusion duration > 24 h.

### 3.2. Secondary Endpoints (Clinical Outcomes)

The median hospital LOS for a course of HDMTX therapy was 4.4 days (IQR 3.8–6.3) (Supplementary Table S2). Courses for ALL reported the shortest median LOS with 3.8 days (IQR 3.0–5.3), while courses for NHL had the longest median LOS (6.8 days, IQR 5.0–15.0). Delay in the subsequent treatment cycle was required in 12.0% of the courses; among the cancers, PCNSL courses were delayed most frequently at 15.6% while only 6% of osteosarcoma courses required a delay. Methotrexate dose reduction in the subsequent course occurred in 18.6% of all courses and was most frequent in PCNSL at 36.6% of the courses, followed by NHL courses at 10.6%. Methotrexate dose omission in the next course occurred in 3% of the total courses, with courses in NHL (6.9%) and PCNSL (3.5%) reporting the highest frequencies. Rehospitalization for toxicity management within 14 days of the start of HDMTX infusion occurred in 5.9% of the courses, and the median LOS for toxicity management was 4.9 days (IQR 2.1–9.5). While higher rates of rehospitalization were found in courses for NHL (11.7%) and osteosarcoma (8.2%) compared to courses for PCNSL (2.8%), median rehospitalization LOS was shortest for osteosarcoma (3.2 days) and longest for PCNSL (15.1 days) and NHL (7.5 days).

### 3.3. Impact of Primary Endpoint Occurrence on Clinical Outcomes and Adverse Events

Courses in which DME occurred required significantly longer delays prior to the next course of treatment compared to courses without DME occurrence (Table 5). Hospital LOS for chemotherapy was significantly longer in courses in which DME occurred (5.3 days, IQR 4.2–9.1) compared to those with no DME (4.2 days, IQR 3.7–6.1). Similarly, courses with AKI and DME + AKI required longer hospital LOS compared to courses without AKI and DME + AKI, respectively. The proportion of courses that required rehospitalization for toxicity was smaller in courses with DME (4.0%) compared to those without DME (6.1%); however, the median LOS of rehospitalization for toxicity management was 1.4 days longer in courses with DME. In contrast, courses with AKI occurrence were observed to have a higher incidence (7.8% vs. 5.5%) of rehospitalization but shorter LOS (3.5 days vs. 5.1 days) than those without AKI. However, these differences in rehospitalization were not significant. Methotrexate dose omission in the subsequent course occurred more frequently in courses that experienced DME, AKI, and DME + AKI compared to those that did not: 9.6% vs. 2.1%, 4.9% vs. 2.7%, and 7.5% vs. 2.8%, respectively. Methotrexate dose reduction in the subsequent course was required more frequently in courses with DME (27.8%) than those without DME (17.3%), whereas it was required less frequently in courses with AKI occurrence (12.5%) compared to those without AKI (19.7%).

Estimated survival rates from the start of HDMTX administration compared by the occurrence of the primary endpoints (Supplementary Table S4) revealed that patients who developed DME had lower 3- and 5-year EFS than those that did not have DME (61.6% vs. 75.3% and 55.9% vs. 71.3%, respectively; log rank *p* < 0.001) as well as lower 3- and 5-year OS (77.4% vs. 88.9% and 76.0% vs. 82.5%, respectively; log rank *p* = 0.003). Patients who experienced DME + AKI also had lower 3- and 5-year EFS compared to those who did not, but this difference was not significant (log rank *p* = 0.095). On the other hand, 3- and 5-year EFS and OS did not differ between patients who had experienced AKI and those who did not.

Courses in which DME occurred reported having higher incidences of Grade 4 neutropenia and thrombocytopenia (28.0% and 29.3%, respectively), compared to courses that did not have DME (7.7% and 9.6%, respectively). Courses with AKI had more frequent incidences of Grades 1 and 2 thrombocytopenia than those without AKI, and courses that experienced DME + AKI reported higher incidences of thrombocytopenia compared to those that did not (Table 6).

### 3.4. Association of Select Concomitant Medications with the Occurrence of Primary Endpoints

Among the 2501 HDMTX courses analyzed, furosemide was administered in 751 (30.0%) courses and proton-pump inhibitors (PPI) in 248 (9.9%) (Table 7). No observable difference in occurrence of primary endpoints was found between courses with and without exposure to PPI. However, among the 384 courses with AKI occurrence, 152 (39.6%) had received furosemide, whereas 28.3% of the 2117 courses without AKI had any exposure to furosemide. While administration of furosemide up to day 3 following the start of HDMTX administration was associated only with AKI, exposure to furosemide on day 4 or later in the course was found to be associated with DME, AKI, and DME + AKI.

## 4. Discussion

High-dose methotrexate is an essential component of treatment for many types of cancers that is safe and effective when administered with meticulous supportive care measures. This study evaluated real-world HDMTX administration practices and outcomes in centers located in Europe to quantify the incidence of DME and AKI. The Registry included a diverse population of both pediatric and adult patients, with the earliest data point collected from a patient diagnosed in 2010 and the last in 2021. Thus, this analysis provides an overview of HDMTX treatment patterns and outcomes of a recent 12-year period in Europe.

The overall incidences of DME and AKI among all treatment courses in this study were 12.1% and 15.4%, respectively, and varied by age and cancer treatment. Occurrence of DME was more frequent in the adult population (≥18 years), whereas AKI occurrence was higher among the pediatric population (Table 3 and Table S1). The higher incidence of AKI in the younger patients may be explained by the AKIN criteria used to define AKI, which includes an increase in Scr of or a greater increase from baseline [21]; this may have resulted in an overestimation of AKI in courses where Scr increased from 0.2 mg/dL at baseline to 0.3 mg/dL at follow-up. A sensitivity analysis comparing AKI incidence according to the ≥50% increase criterion vs. an absolute Scr increase of 0.3 mg/dL criterion in courses would possibly result in a lower incidence of AKI in the pediatric population. Among the cancer types, AKI was most frequent in courses for ALL (21.0%), but despite pediatric patients comprising 89% ALL, a higher proportion of ALL courses in adults (35.8%) were reported to have AKI compared to courses in children (19.2%), indicating age as a potential risk factor for AKI in ALL. In osteosarcoma and NHL, however, AKI incidences were comparable between children and adults despite differences in age distribution within the cancer. Also, while differences in DME and AKI incidence were observed among different doses and infusion durations (Table 4), no definite correlation was elucidated between methotrexate dose and infusion duration with the primary endpoints. The lack of observable associations is likely due to the different HDMTX treatment protocols administered to patients of the different cancers, in which the age distribution of patients also varied. These findings suggest that neither age nor treatment-related factors can predict the occurrence of DME or AKI a priori with sufficient sensitivity and specificity to be clinically useful and underscore the importance of vigilant monitoring of MTXc for early detection with validated tools such as MTXPK.org [18,23].

Occurrence of DME was associated with longer hospital LOS, longer delays before starting the next cycle of treatment, and higher rates of Grade 4 neutropenia and thrombocytopenia (Table 6), both of which may have contributed to the higher rates of methotrexate dose reduction and dose omission in subsequent courses. Further, patients in whom DME occurred at any point during their treatment were found to have 13.7% lower 3-year EFS and 15.4% lower 5-year EFS, as well as lower OS compared to those who did not experience DME (Supplementary Table S4). These findings demonstrate the negative impact of DME on HDMTX treatment outcomes and highlight the importance of supportive care measures for its prevention and prompt management. While leucovorin rescue was administered in 99.6% of ALL and 95.1% of osteosarcoma courses, it was provided in only 88% of CNS cancer and 93.3% of NHL courses, both of which were composed of predominantly adult patients. The reason for the lack of leucovorin rescue in these courses could not be determined as no clinical notes were reported in the Registry, but this does serve as a reminder to provide sufficient leucovorin rescue, especially in older patients who may be more susceptible to toxicities [4,25]. It is noteworthy that although DME was found in 302 courses, glucarpidase was administered in only 8 courses in 8 different individuals. The missed opportunities for glucarpidase administration likely reflect the timing of the European Medicines Agency (EMA) authorization for glucarpidase approval in January 2022 [26], a considerable amount of time after the period of data collection for the Registry, and the eight cases of glucarpidase administration were most likely from the compassionate-use named patient program or available institutional funding. Nonetheless, the very small proportion of DME cases that received glucarpidase reflects the opportunities that could have resulted in potentially improved clinical outcomes had glucarpidase been provided per the prescription guidelines. A follow-up analysis is currently underway describing the glucarpidase cases and missed opportunities from the Registry.

The Registry collected data on concomitant medications; two non-chemotherapeutic agents, the loop diuretic furosemide and PPIs, were reported in this study based on their potentially negative impact on renal function [27,28,29,30]. Reported use of PPI and furosemide was 10% and 30% of all courses (Table 7), respectively, with both administered most frequently in ALL, at 12.4% and 38.9%, respectively, compared to the other cancers. Although PPI was not associated with either AKI or DME in this population, furosemide use at any time point was associated with AKI, and furosemide use on day 4 or later after start of HDMTX administration was found to be associated with DME. It was a bit alarming that furosemide was administered in 30% of all HDMTX courses and close to 40% of courses for ALL, and this finding serves as a reminder that clinicians should be aware of medications and drug interactions that can worsen the nephrotoxicity associated with HDMTX. It is noted that the other agents administered as a component of multiagent chemotherapy for these cancers carried the risk of adverse events, which may have led to poor clinical outcomes, but it was beyond the scope of this study to parse out their individual impact.

There are several limitations in this study. The various treatment regimens for the different cancers and varying age distribution of patients within the cancer types rendered elucidation of specific factors (e.g., methotrexate dose, infusion duration) to predict the development of DME and AKI very difficult. Further, the relatively smaller number of osteosarcoma patients included in this Registry may have under-represented the outcomes of the real-world setting and resulted in lower incidences of DME and AKI than those reported in the literature [15,31,32]. More granular analyses by age group and treatment factors within each cancer type would allow for more focused information to determine potential predictors of HDMTX nephrotoxicity. It must be noted that this study was intended as a descriptive study rather than providing a causal analysis between independent variables and DME and AKI; a causal pathway was not set up a priori, and thus, analyses such as multivariate regression modeling were beyond the scope of this paper. This study, however, was able to provide a broad overview of HDMTX treatment and its association with clinical outcomes in four cancer categories that can serve as a foundation for more detailed investigations. Supportive therapy reported in this study was limited to leucovorin rescue and glucarpidase administration, and measures including hyperhydration and urine alkalinization (with pH monitoring), which are essential for preventing nephrotoxicity, were not presented in this study. While these data were collected in the Registry, data recording errors and the large number of missing data precluded them from being included in this analysis. A separate analysis examining the data collected on all supportive care measures and outcomes by a cancer type will be conducted to fill this gap. Similarly, the large number of missing data for laboratory findings, particularly for neutropenia and thrombocytopenia (Table 6), precludes making definite associations with DME or AKI. Also, although the focus of this study was on the clinical impact of HDMTX, the majority of the treatment protocols employed polychemotherapy that incorporates other potentially nephrotoxic agents, which may have contributed to the development of DME and AKI. Lastly, while this study provides real-world data on HDMTX treatment and outcomes in patients receiving treatment in European countries under ideal conditions and well-funded healthcare systems, it does not reflect treatment conditions in lower and middle-income countries (LMICs), where patients often receive less-than-ideal treatment doses due to limited access to supportive care, such as obtaining methotrexate levels in a timely manner or access to glucarpidase [33,34,35,36]. The findings of this study are still valuable as they demonstrate the importance of vigilant monitoring of MTXc to prevent nephrotoxicity even in high-income countries, such that measures to provide better access to supportive care can be promoted to governing bodies in LMICs.

## 5. Conclusions

High-dose methotrexate is a very effective and safe treatment when provided with meticulous supportive therapy. Despite supportive measures, administration of efficacious doses of HDMTX can lead to AKI and DME, and no single or combination of patient or treatment factors was found to reliably predict their occurrence. Thus, diligent monitoring of methotrexate concentration with sensitive tools such as MTXPK.org is paramount for early detection and prompt management of nephrotoxicity in all settings where HDMTX treatment is administered.

## Figures and Tables

**Table 1:** Demographic characteristics of patients by cancer type.

	All Patients (N = 588)	ALL (N = 283)	PCNSL (N = 158)	NHL (N = 108)	Osteosarcoma (N = 29)	Other CNSC (N = 10)
**Age**, years						
Median	16.4	6.0	66.4	57.3	15.2	2.7
Q1, Q3	5.6, 62.2	3.5, 13.3	58.0, 75.4	12.3, 69.9	10.6, 17.4	1.9, 7.9
Range	0.2–89.5	0.2–82.5	19.7–89.5	3.5–83.3	4.4–45.5	0.5–69.7
**Age group**, years						
<12, n (%)	248 (42.2)	205 (72.4)	0	26 (24.1)	9 (31.0)	8 (80.0)
≥12 to <18, n (%)	61 (10.4)	39 (13.8)	0	7 (6.5)	14 (48.3)	1 (10.1)
≥18 to <65, n (%)	150 (25.5)	33 (11.7)	73 (46.2)	38 (35.2)	6 (20.7)	0
≥65, n (%)	129 (21.9)	6 (2.1)	85 (53.8)	37 (34.3)	0	1 (10.0)
**Sex**						
Female, n (%)	276 (46.9)	136 (48.1)	86 (54.4)	41 (38.0)	9 (31.0)	4 (40.0)
Male, n (%)	312 (53.1)	147 (51.9)	72 (45.6)	67 (62.0)	20 (69.0)	6 (60.0)
**Race**						
Asian, n (%)	9 (1.6)	6 (2.1)	0	1 (0.9)	1 (3.4)	1 (10.0)
Black, n (%)	3 (0.5)	0	0	2 (1.9)	1 (3.4)	0
White, n (%)	273 (46.4)	184 (65.0)	13 (8.2)	49 (45.4)	20 (69.0)	7 (70.0)
Other, n (%)	12 (2.0)	10 (3.5)	0	1 (0.9)	1 (3.4)	0
Unknown, n (%)	291 (49.5)	83 (29.4)	145 (91.8)	55 (50.9)	6 (20.7)	2 (20.0)
**Investigational Site Country**						
France, n (%)	95 (16.2)	0	91 (57.6)	4 (3.7)	0	0
Germany, n (%)	103 (17.5)	59 (20.8)	3 (1.9)	32 (29.6)	4 (13.8)	5 (50.0)
Italy, n (%)	135 (23.0)	81 (28.6)	52 (32.9)	2 (1.9)	0	0
Spain, n (%)	203 (34.5)	137 (48.4)	12 (7.6)	49 (45.4)	4 (14.8)	1 (10.0)
United Kingdom, n (%)	52 (8.8)	6 (2.1)	0	21 (19.4)	21 (72.4)	4 (40.0)

Abbreviations: ALL, acute lymphoblastic leukemia; CNSC, central nervous system cancer; NHL, non-Hodgkin lymphoma; PCNSL, primary CNS lymphoma.

**Table 2:** Characteristics of high-dose methotrexate administration by cancer type.

	All Courses (N = 2501)	ALL (N = 988)	PCNSL (N = 853)	NHL (N = 349)	Osteosarcoma (N = 267)	Other CNSC (N = 44)
**Age group**, years						
Pediatric (<18), n (%)	1288 (51.5)	877 (88.8)	0	132 (37.8)	236 (88.4)	43 (97.7)
Adult (≥18), n (%)	1213 (48.5)	111 (11.2)	853 (100)	217 (62.2)	31 (11.6)	1 (2.3)
**Sex**						
Female, n (%)	1229 (49.1)	487 (49.3)	513 (60.1)	122 (35.0)	82 (30.7)	25 (56.8)
Male, n (%)	1272 (50.9)	501 (50.7)	340 (39.9)	227 (65.0)	185 (69.3)	19 (43.2)
**HDMTX dose standard**, g/m^2^						
Median	3.5	5.0	2.9	3.0	11.8	5.1
Q1, Q3	2.7, 5.0	4.8, 5.0	2.1, 3.0	1.5, 3.4	11.2, 12.0	5.0, 8.3
Range	0.3–13.5	0.4–10.2	0.5–3.8	0.3–8.8	3.0–13.5	3.0–11.7
**HDMTX dose category**, g/m^2^						
<2.5, n (%)	572 (22.9)	103 (10.4)	339 (39.7)	130 (37.2)	0	0
≥2.5 to <4, n (%)	772 (30.9)	95 (9.6)	514 (60.3)	159 (45.6)	3 (1.1)	1 (2.3)
≥4 to <6.5, n (%)	847 (33.9)	783 (79.3)	0	28 (8.0)	11 (4.1)	25 (56.8)
≥6.5 to <10, n (%)	67 (2.7)	6 (0.6)	0	32 (9.2)	12 (4.5)	17 (38.6)
≥10, n (%)	243 (9.7)	1 (0.1)	0	0	241 (90.3)	1 (2.3)
**Infusion duration**, hours						
Median	7.0	24.0	6.0	4.0	4.0	24.0
Q1, Q3	4.0, 24.0	24.0, 24.0	4.0, 6.0	3.0, 24.0	4.0, 4.0	23.0, 24.0
Range	1.0–40.0	4.0–40.0	1.0–28.0	2.0–39.0	3.0–23.0	2.0–29.0
**Infusion duration categories,** hours						
<5, n (%)	709 (28.3%)	5 (0.5)	246 (28.8)	191 (54.7)	262 (98.1)	5 (11.4)
≥5 to ≤10, n (%)	620 (24.8%)	4 (0.4)	589 (69.1)	23 (6.6)	3 (1.1)	1 (2.3)
>10 to <24, n (%)	173 (6.9%)	134 (13.6)	5 (0.6)	21 (6.0)	2 (0.7)	11 (25.0)
≥24, n (%)	999 (39.9%)	845 (85.5)	13 (1.5)	114 (32.7)	0	27 (61.4)
**Infusion duration category**, hours						
Short ≤ 10, n (%)	1329 (53.1)	9 (0.9)	835 (97.9)	214 (61.3)	265 (99.3)	6 (13.6)
Long > 10 n (%)	1172 (46.9)	979 (99.1)	12 (2.1)	135 (38.7)	2 (0.7)	38 (86.4)
**Loading methotrexate dose per administration**, mg						
Median	494.1	495.2	492.5	286.0	NA	508.8
Q1, Q3	466.9, 502.2	475.5, 503.2	477.7, 500.6	101.0, 488.8	NA	495.2, 585.5
Range	26.4–893.6	26.4–797.8	143.5–537.6	41.9–511.9	NA	491.5–893.6
Missing, n	1308	60	722	243	267	16
**Leucovorin rescue**, courses						
No, n (%)	116 (4.6%)	4 (0.4)	54 (6.3)	44 (12.6)	13 (4.9%)	1 (2.3)
Yes, n (%)	2385 (95.4%)	984 (99.6)	799 (93.7)	305 (87.4)	254 (95.1%)	43 (97.7)
95% CI (%)	94.6–96.15	98.97–99.89	91.82–95.21	83.45–90.69	91.82–97.38	87.98–99.94
**Glucarpidase administration,** courses						
No, n (%)	2493 (99.7%)	985 (99.7)	848 (99.4)	349 (100)	267 (100)	44 (100)
Yes, n (%)	8 (0.3%)	3 (0.3)	5 (0.6)	0	0	0

Abbreviations: ALL, acute lymphoblastic leukemia; CNSC, central nervous system cancer; HDMTX, high-dose methotrexate; NHL, non-Hodgkin lymphoma; PCNSL, primary CNS lymphoma.

**Table 3:** Primary endpoints by age group and cancer type (N = 2501).

	All Courses (N = 2501)	Age < 18 (N = 1288)	Age ≥ 18 (N = 1213)	ALL (N = 983)	PCNSL (N = 853)	NHL (N = 349)	Osteosarcoma (N = 267)	Other CNSC (N = 44)
**DME**								
No, n (%)	2199 (87.9)	1212 (94.1)	987 (81.4)	907 (91.8)	698 (81.8)	289 (82.8)	263 (98.5)	42 (95.5)
Yes n (%)	302 (12.1)	76 (5.9)	226 (18.6)	81 (8.2)	155 (18.2)	60 (17.2)	4 (1.5)	2 (4.5)
95% CI (%)	10.82–13.42	4.56–7.33	16.48–20.94	6.56–10.09	15.64–20.93	13.38–21.57	0.41–3.79	0.56–15.47
**AKI**								
No, n (%)	2117 (84.6)	1076 (83.5)	1041 (85.8)	781 (79.0)	768 (90.0)	283 (81.1)	248 (92.9)	37 (84.1)
Yes n (%)	384 (15.4)	212 (16.5)	172 (14.2)	207 (21.0)	85 (10.0)	66 (18.9)	19 (7.1)	7 (15.9)
95% CI (%)	13.96–16.83	14.47–18.60	12.26–16.27	18.45–23.62	8.04–12.17	14.94–23.42	4.34–10.89	6.64–30.07
**Severe AKI ^1^**								
No, n (%)	2423 (96.9)	1234 (95.8)	1189 (98.0)	935 (94.6)	842 (98.7)	340 (97.4)	264 (98.9)	42 (95.5)
Yes n (%)	78 (3.1)	54 (4.2)	24 (2.0)	53 (5.4)	11 (1.3)	9 (2.6)	3 (1.1)	2 (4.5)
95% CI (%)	2.47–3.88	3.16–5.44	1.27–2.93	4.04–6.96	0.65–2.30	1.19–4.84	0.23–3.25	0.56–15.47
**DME + AKI**								
No, n (%)	2395 (95.8)	1251 (97.1)	1144 (94.3)	944 (95.5)	817 (95.8)	325 (93.1)	265 (99.3)	44 (100)
Yes n (%)	106 (4.2%)	37 (2.9)	69 (5.7)	44 (4.5)	36 (4.2)	24 (6.9)	2 (0.7)	0
95% CI (%)	3.48–5.10	2.03–3.94	4.45–7.14	3.25–5.93	2.97–5.80	4.46–10.06	0.09–2.68	0.00–0.00

Abbreviations: AKI, acute kidney injury; ALL, acute lymphoblastic leukemia; CNSC, central nervous system cancer; DME, delayed methotrexate elimination; NHL, non-Hodgkin lymphoma; PCNSL, primary CNS lymphoma. ^1^ Acute Kidney Injury Network (AKIN) criteria stage 2 or 3.

**Table 4:** Occurrence of primary endpoints by methotrexate dose and infusion duration.

	Dose, g/m^2^					Infusion Duration, Hours			
	<2.5 (N = 572)	≥2.5 to <4 (N = 772)	≥4 to <6.5 (N = 847)	≥6.5 to <10 (N = 67)	>10 (N = 243)	<5 (N = 709)	≥5 to <10 (N = 620)	≥10 to <24 (N = 173)	≥24 (N = 999)
**DME**									
No, n (%)	485 (84.5%)	616 (79.8%)	793 (93.6%)	64 (95.5%)	241 (99.2%)	558 (78.7%)	566 (91.3%)	163 (94.2%)	912 (91.3%)
Yes, n (%)	87 (15.2%)	156 (20.2%)	54 (6.4%)	3 (4.5%)	2 (0.8%)	151 (21.3%)	54 (8.7%)	10 (5.8%)	87 (8.7%)
95% CI (%)	12.37–18.42	17.43–23.22	4.83–8.24	0.93–12.53	0.10–2.94	18.34–24.50	6.61–11.21	2.81–10.37	7.03–10.63
**AKI**									
No, n (%)	499 (87.2%)	662 (85.8%)	673 (79.5%)	57 (85.1%)	226 (93.0%)	613 (86.5%)	576 (92.9%)	149 (86.1%)	779 (78.0%)
Yes, n (%)	73 (12.8%)	110 (14.2%)	174 (20.5%)	10 (14.9%)	17 (7.0%)	96 (13.5%)	44 (7.1%)	24 (13.9%)	220 (22.0%)
95% CI (%)	10.14–15.78	11.86–16.92	17.87–23.42	7.40–25.74	4.12–10.96	11.11–16.28	5.20–9.41	9.10–19.94	19.49–24.72
**Severe AKI ^1^**									
No, n (%)	563 (98.4%)	756 (97.9%)	802 (96.1%)	61 (91.0%)	241 (99.2%)	695 (98.0%)	613 (98.9%)	171 (98.8%)	944 (94.5%)
Yes, n (%)	9 (1.6%)	16 (2.1%)	45 (5.3%)	6 (9.0%)	2 (0.8%)	14 (2.0%)	7 (1.1%)	2 (1.2%)	55 (5.5%)
95% CI (%)	0.72–2.97	1.19–3.34	3.90–7.04	3.36–18.48	0.10–2.94	1.08–3.29	0.46–2.31	0.14–4.11	4.17–7.11
**DME + AKI**									
No, n (%)	543 (94.9%)	731 (94.7%)	814 (96.1%)	65 (97.0%)	242 (99.6%)	668 (94.2%)	606 (97.7%)	169 (97.7%)	952 (95.3%)
Yes, n (%)	29 (5.1%)	41 (5.3%)	33 (3.9%)	2 (3.0)	1 (0.4%)	41 (5.8%)	14 (2.3%)	4 (2.3%)	47 (4.7%)
95% CI (%)	3.42–7.20	3.84–7.14	2.70–5.43	0.36–10.37	0.01–2.27	4.18–7.76	1.24–3.76	0.63–5.81	3.48–6.21

Abbreviations: AKI, acute kidney injury; DME, delayed methotrexate elimination. ^1^ Acute Kidney Injury Network (AKIN) criteria stage 2 or 3.

**Table 5:** Cross-tabulations for clinical outcomes by the occurrence of primary endpoints.

	All Courses (N = 2501)	DME Yes (N = 302)	DME No (N = 2199)	AKI Yes (N = 384)	AKI No (N = 2117)	DME + AKI Yes (N = 106)	DME + AKI No (N = 2395)
**Delay of subsequent cycle**, days							
Median	17.9	25.8	16.0	18.1	17.7	23.0	17.0
Q1, Q3	14.0, 28.0	16.7, 32.0	14.0, 27.3	14.0, 28.0	14.0, 28.0	15.0, 30.0	14.0, 27.9
Range	5.9–959.7	7.0–281.8	5.9–959.7	6.0–228.9	5.9–959.7	13.9–97.0	5.9–959.7
Missing	613	106	507	105	508	37	576
*Χ*^2^ *p*-value		<0.001		0.115		<0.001	
**Hospital LOS for therapy**, days							
Median	4.4	5.3	4.2	5.2	4.3	6.7	4.3
Q1, Q3	3.8, 6.3	4.2, 9.1	3.7, 6.1	3.9, 8.1	3.8, 6.0	5.0, 10.31	3.8, 6.2
Range	0.0–375.2	0.0–375.2	0.0–201.8	0.3–375.2	0.0–373.5	1.9–375.2	0.0–373.5
*Wilcoxon rank-sum p*-value		<0.001		<0.001		<0.001	
**Rehospitalization for toxicity**							
No, n (%)	2354 (94.1%)	290 (96.0%)	2064 (93.9%)	354 (92.2%)	2000 (94.5%)	99 (93.4%)	2255 (94.2%)
Yes, n (%)	147 (5.9%)	12 (4.0%)	135 (6.1%)	30 (7.8%)	117 (5.5%)	7 (6.6%)	140 (5.8%)
95% CI (%)	4.99–6.87	2.07–6.84	5.17–7.23	5.33–10.97	4.59–6.59	2.70–13.13	4.94–6.86
*Χ*^2^ *p*-value		0.134		0.080		0.745	
**Rehospitalization LOS**, days							
Median	4.9	6.3	4.9	3.5	5.1	2.0	5.0
Q1, Q3	2.1, 9.5	1.8, 11.7	2.1, 8.9	1.5, 8.0	2.9, 11.2	1.3, 5.9	2.3, 10.1
Range	0.0–172.1	0.1–39.4	0.0–172.1	0.1–119.9	0.0–172.1	0.1–10.6	0.0–172.1
*Wilcoxon rank-sum p*-value		0.860		0.630		0.105	
**MTX dose reduction in next course**							
No, n (%)	2036 (81.4%)	218 (72.2%)	1818 (82.7%)	336 (87.5%)	1700 (80.3%)	89 (84.0%)	1947 (81.3%)
Yes, n (%)	465 (18.6%)	84 (27.8%)	381 (17.3%)	48 (12.5%)	417 (19.7%)	17 (16.0%)	448 (18.7%)
95% CI (%)	17.09–20.17	22.84–33.24	15.77–18.97	9.36–16.23	18.02–21.46	9.63–24.43	17.16–20.33
*Χ*^2^ *p*-value		<0.001		<0.001		0.490	
**MTX dose omission in next course**							
No, n (%)	2425 (97.0%)	273 (90.4%)	2152 (97.9%)	365 (95.1%)	2060 (97.3%)	98 (92.5%)	2327 (97.2%)
Yes, n (%)	76 (3.0%)	29 (9.6%)	47 (2.1%)	19 (4.9%)	57 (2.7%)	8 (7.5%)	68 (2.8%)
95% CI (%)	2.40–3.79	6.53–13.50	1.57–2.83	3.00–7.62	2.05–3.47	3.31–14.33	2.21–3.59
*Χ*^2^ *p*-value		<0.001		0.018		0.006	

**Table 6:** Cross-tabulations for adverse events by the occurrence of primary endpoints.

	All Courses (N = 2501)	DME Yes (N = 302)	DME No (N = 2199)	AKI Yes (N = 384)	AKI No (N = 2117)	DME + AKI Yes (N = 106)	DME + AKI No (N = 2395)
**Transaminitis**, CTCAE grade							
0	1089 (60.9%)	162 (57.7%)	927 (61.5%)	176 (55.0%)	913 (62.2%)	63 (63.0%)	1026 (60.7%)
1	584 (32.6%)	95 (33.8%)	489 (32.4%)	120 (37.5%)	464 (31.6%)	31 (31.0%)	553 (32.7%)
2	60 (3.4%)	10 (3.6%)	50 (3.3%)	11 (3.4%)	49 (3.3%)	3 (3.0%)	57 (3.4%)
3	53 (3.0%)	14 (5.0%)	39 (2.6%)	12 (3.8%)	41 (2.8%)	3 (3.0%)	50 (3.0%)
4	3 (0.2%)	0 (0.0%)	3 (0.2%)	1 (0.3%)	2 (0.1%)	0 (0.0%)	3 (0.2%)
Missing, n	712	21	691	64	648	6	706
95% CI (%)	30.47–34.87	28.30–39.67	30.07–34.86	32.18–43.06	29.21–34.03	22.13–41.03	30.51–35.04
*p*-value		0.207		0.176		0.984	
**Hyperbilirubinemia**, CTCAE grade							
0	1306 (83.3%)	145 (82.9%)	1161 (83.3%)	174 (77.7%)	1132 (84.2%)	41 (74.5%)	1265 (83.6%)
1	168 (10.7%)	21 (12.0%)	147 (10.6%)	33 (14.7%)	135 (10.0%)	11 (20.0%)	157 (10.4%)
2	87 (5.5%)	8 (4.6%)	79 (5.7%)	15 (6.7%)	72 (5.4%)	2 (3.6%)	85 (5.6%)
3	5 (0.3%)	1 (0.6%)	4 (0.3%)	2 (0.9%)	3 (0.2%)	1 (1.8%)	4 (0.3%)
4	2 (0.1%)	0 (0.0%)	2 (0.1%)	0 (0.0%)	2 (0.1%)	0 (0.0%)	2 (0.1%)
Missing, n	933	127	806	160	773	51	882
95% CI (%)	9.23–12.35	7.58–17.76	8.99–12.29	10.36–20.06	8.49–11.78	10.43–32.97	8.89–12.02
*p*-value		0.863		0.073		0.048	
**Neutropenia**, CTCAE grade							
0	49 (5.7%)	6 (3.8%)	43 (6.2%)	5 (5.1%)	44 (5.8%)	1 (2.6%)	48 (5.9%)
1	532 (62.3%)	75 (47.8%)	457 (65.6%)	57 (57.6%)	475 (62.9%)	21 (53.8%)	511 (62.7%)
2	83 (9.7%)	15 (9.6%)	68 (9.8%)	10 (10.1%)	73 (9.7%)	6 (15.4%)	77 (9.4%)
3	92 (10.8%)	17 (10.8%)	75 (10.8%)	12 (12.1%)	80 (10.6%)	4 (10.3%)	88 (10.8%)
4	98 (11.5%)	44 (28.0%)	54 (7.7%)	15 (15.2%)	83 (11.0%)	7 (17.9%)	91 (11.2%)
Missing, n	1647	145	1502	285	1362	67	1580
95% CI (%)	58.95–65.56	39.75–55.88	61.91–69.09	47.23–67.45	59.36–66.37	37.18–69.91	59.28–66.03
*p*-value		<0.001		0.733		0.401	
**Thrombocytopenia**, CTCAE grade							
0	660 (52.4%)	67 (29.3%)	593 (57.5%)	96 (41.2%)	564 (54.9%)	20 (26.3%)	640 (54.1%)
1	308 (24.4%)	60 (26.2%)	248 (24.1%)	71 (30.5%)	237 (23.1%)	27 (35.5%)	281 (23.7%)
2	67 (5.3%)	20 (8.7%)	47 (4.6%)	27 (11.6%)	40 (3.9%)	10 (13.2%)	57 (4.8%)
3	59 (4.7%)	15 (6.6%)	44 (4.3%)	12 (5.2%)	47 (4.6%)	5 (6.6%)	54 (4.6%)
4	166 (13.2%)	67 (29.3%)	99 (9.6%)	27 (11.6%)	139 (13.5%)	14 (18.4%)	152 (12.8%)
Missing, n	1241	73	1168	151	1090	30	911
95% CI (%)	22.09–26.92	20.63–32.40	21.47–26.78	24.63–36.82	20.53–25.78	24.88–47.34	21.34–26.26
*p*-value		<0.001		<0.001		<0.001	

**Table 7:** Cross-tabulations for medications by the occurrence of primary endpoints.

	All Courses (N = 2501)	DME Yes (N = 302)	DME No (N = 2199)	AKI Yes (N = 384)	AKI No (N = 2117)	DME + AKI Yes (N = 106)	DME + AKI No (N = 2395)
**Proton-pump inhibitor** **exposure during course**							
No, n (%)	2253 (90.1%)	277 (91.7%)	1976 (89.9%)	341 (88.8%)	1912 (90.3%)	97 (91.5%)	2156 (90.0%)
Yes, n (%)	248 (9.9%)	25 (8.3%)	223 (10.1%)	43 (11.2%)	205 (9.7%)	9 (8.5%)	239 (10.0%)
95% CI (%)	8.77–11.15	5.43–11.98	8.91–11.48	8.22–14.79	8.46–11.02	3.96–15.51	8.81–11.25
*p*-value		0.310		0.361		0.616	
**Furosemide exposure (any) during course**							
No, n (%)	1750 (70.0%)	212 (70.2%)	1538 (69.9%)	232 (60.4%)	1518 (71.7%)	66 (62.3%)	1684 (70.3%)
Yes, n (%)	751 (30.0%)	90 (29.8%)	661 (30.1%)	152 (39.6%)	599 (28.3%)	40 (37.7%)	711 (29.7%)
95% CI (%)	28.24–31.87	24.70–35.31	28.15–32.02	34.66–44.67	26.38–30.27	28.50–47.67	27.86–31.56
*p*-value		0.927		<0.001		0.077	
**Early furosemide (up to day 3) exposure during course**							
No, n (%)	1784 (71.3%)	217 (71.9%)	1567 (71.3%)	238 (62.0%)	1546 (73.0%)	69(65.1%)	1715 (71.6%)
Yes, n (%)	717 (28.7%)	85 (28.1%)	632 (28.7%)	146 (38.0%)	571 (27.0%)	37 (34.9%)	680 (28.4%)
95% CI (%)	26.90–30.49	23.14–33.58	26.86–30.68	33.14–43.08	25.09–28.92	25.90–44.78	26.59–30.24
*p*-value		0.830		<0.001		0.147	
**Late furosemide (day 4 or later) exposure**							
No, n (%)	2281 (91.2%)	253 (83.8%)	2028 (92.2%)	301 (78.4%)	1980 (93.5%)	76 (71.7%)	2205 (92.1%)
Yes, n (%)	220 (8.8%)	49 (16.2%)	171 (7.8%)	83 (21.6%)	137 (6.5%)	30 (28.3%)	190 (7.9%)
95% CI (%)	7.72–9.98	12.25–20.88	6.69–8.98	17.60–26.07	5.46–7.60	19.98–37.88	6.88–9.09
*p*-value		<0.001		<0.001		<0.001	

## Data Availability

Restrictions apply to the availability of the datasets, as the Registry data were obtained from BTG and are available from the authors with the permission of BTG.

## Supplementary Materials

The following supporting information can be downloaded at: https://www.mdpi.com/article/10.3390/cancers18010124/s1. Table S1. Primary endpoints by cancer type and age group (N=2501); Table S2. Incidence and characteristics of secondary endpoints by cancer type; Table S3. Three- and 5-year event-free survival and overall survival estimates by cancer type; Table S4. Event-free and overall survival estimates by occurrence of the primary endpoints; Table S5. Total number of courses included in the Registry by investigational site (N = 3165); Figure S1. Kaplan-Meier estimates for EFS by cancer type from start of HDMTX administration; Figure S2. Kaplan-Meier estimates for OS by cancer type from start of HDMTX administration
