# Correction: Pourroy et al. High-Dose Methotrexate at All Ages: Safety, Efficacy, and Outcomes from the HDMTX European Registry. *Cancers* 2026, *18*, 124

**DOI:** 10.3390/cancers18060941

**Published:** 2026-03-13

**Authors:** Bertrand Pourroy, Maria D. Aumente, Christian Koenecke, Martin Stanulla, Andrés J. M. Ferreri, Thais M. Carillo, Madhumita Dandapani, Timothy A. Ritzmann, Pere Barba, Etienne Chatelut, Katrina M. Ingley, Emma Morris, Elisabeth Schorb, Sven Liebig, Stefan Schwartz, Scott C. Howard, Ryan Combs, Nicolás Tentoni, Jennifer Lowe, Gabriela Villanueva, Claudia Sampor, Miriam Hwang, Carmelo Rizzari

**Affiliations:** 1 Oncopharma Unit, Hôpitaux Universitaires de Marseille La Timone, 13005 Marseille, France; 2 Pharmacy Service, Reina Sofia University Hospital, 14004 Cordoba, Spain; 3 Department of Pediatric Hematology and Oncology, University of Hannover, 30167 Hannover, Germany; 4 Lymphoma Unit, IRCCS San Raffaele Scientific Institute, 20132 Milano, Italy; 5 Pediatric Oncology & Hematology, Hospital Universitari Vall d’Hebron, 08035 Barcelona, Spain; 6 Department of Pediatrics Oncology/Haematology, University of Nottingham, Nottingham NG5 1PB, UK; 7 Children’s Brain Tumour Research Centre, University of Nottingham, Nottingham NG7 2UH, UK; 8 Hematology Department, Hospital Universitari Vall d’Hebron, 08035 Barcelona, Spain; 9 Department of Pharmacology, Institut Universitaire du Cancer-Oncopole, 31059 Toulouse, France; 10 Sarcoma Department, University College London Hospital, London NW1 2PB, UK; 11 Pharmacy Cancer Services, University College London Hospital, London NW1 2PB, UK; 12 Comprehensive Cancer Center, University of Freiburg, 79106 Freiburg, Germany; 13 Department of Hematology, Oncology and Tumor Immunology, Charite Universitätsmedizin, 10117 Berlin, Germany; 14 Resonance, Memphis, TN 38104, USA; 15 Global Development Office, Hospital Sant Joan de Deu, 08950 Barcelona, Spain; 16 Department of Pediatrics, University of Milano-Bicocca, 20126 Milan, Italy

**Text Correction**

There was an error in the original publication [1]. “Percentages presented in the text in the seventh (36.8% vs. 19.0%) and eighth sentences (9.6%; 2.1%) were errors and did not match those in Table 5 of which they were referenced”.

A correction has been made to 3. Results section, *3.3. Impact of Primary Endpoint Occurrence on Clinical Outcomes and Adverse Events* subsection, first paragraph, seventh and eighth sentences:

Courses in which DME occurred required significantly longer delays prior to the next course of treatment compared to courses without DME occurrence (Table 5). Hospital LOS for chemotherapy was significantly longer in courses in which DME occurred (5.3 days, IQR 4.2–9.1) compared to those with no DME (4.2 days, IQR 3.7–6.1). Similarly, courses with AKI and DME + AKI required longer hospital LOS compared to courses without AKI and DME + AKI, respectively. The proportion of courses that required rehospitalization for toxicity was smaller in courses with DME (4.0%) compared to those without DME (6.1%); however, the median LOS of rehospitalization for toxicity management was 1.4 days longer in courses with DME. In contrast, courses with AKI occurrence were observed to have a higher incidence (7.8% vs. 5.5%) of rehospitalization but shorter LOS (3.5 days vs. 5.1 days) than those without AKI. However, these differences in rehospitalization were not significant. Methotrexate dose omission in the subsequent course occurred more frequently in courses that experienced DME, AKI, and DME + AKI compared to those that did not: 9.6% vs. 2.1%, 4.9% vs. 2.7%, and 7.5% vs. 2.8%, respectively. Methotrexate dose reduction in the subsequent course was required more frequently in courses with DME (27.8%) than those without DME (17.3%), whereas it was required less frequently in courses with AKI occurrence (12.5%) compared to those without AKI (19.7%). 

The authors apologize for any inconvenience caused and state that the scientific conclusions are unaffected. This correction was approved by the Academic Editor. The original publication has also been updated.
