# Supplementary material for: High-Dose Methotrexate at All Ages: Safety, Efficacy, and Outcomes from the HDMTX European Registry
Source: Cancers (Basel). 2025 Dec 30;18(1):124. doi: 10.3390/cancers18010124 (PMC12784913; doi:10.3390/cancers18010124)
Supplement: Supplementary file 1 [file cancers-18-00124-s001.zip › Table S1.pdf]

Table S1. Primary endpoints by cancer type and age group (N=2501)

	**ALL**		**PCNSL**		**NHL**		**Osteosarcoma**		**Other CNS cancers**	
	< 18 years (N=877)	≥ 18 years (N=111)	< 18 years (N=0)	≥ 18 years (N=853)	< 18 years (N=132)	≥ 18 years (N=217)	< 18 years (N=236)	≥ 18 years (N=31)	< 18 years (N=43)	≥ 18 years (N=1)
**DME**										
No, n (%)	815 (92.9)	92 (82.9)		698 (81.8)	122 (92.4)	167 (77.0)	233 (98.7)	30 (96.8)	42 (97.7)	0
Yes, n (%)	62 (7.1)	19 (17.1)		155 (18.2)	10 (7.6)	50 (23.0)	3 (1.3)	1 (3.2)	1 (2.3)	1 (100)
95% CI (%)	5.46 - 8.97	10.63 - 25.43		15.64 - 20.93	3.69 - 13.49	17.61 - 29.22	0.26 - 3.67	0.08 - 16.70	0.06 - 12.29	2.50 - 100.00
**AKI**										
No, n (%)	709 (80.8)	72 (64.9)		768 (90.0)	112 (84.8)	171 (78.8)	219 (92.8%	29 (93.5)	36 (83.7)	1 (100)
Yes, n (%)	168 (19.2)	39 (35.1)		85 (10.0)	3 (2.3)	6 (2.8)	17 (7.2)	2 (6.5)	7 (16.3)	0
95% CI (%)	16.60 - 21.92	26.31 - 44.77		8.04 - 12.17	0.47 - 6.50	1.02 - 5.92	4.25 - 11.28	0.79 - 21.42	6.81 - 30.70	0.00 - 0.00
**Severe AKI**										
No, n (%)	831 (94.8)	104 (93.7)		842 (98.7)	129 (97.7)	211 (97.2)	233 (98.7)	31 (100)	41 (95.3)	1 (100)
Yes, n (%)	46 (5.2)	7 (6.3)		11 (1.3)	3 (2.3)	6 (2.8)	3 (1.3)	0	2 (4.7%)	0
95% CI (%)	3.87 - 6.93	2.57 - 12.56		0.65 - 2.30	0.47 - 6.50	1.02 - 5.92	0.26 - 3.67	0.00 - 0.00	0.57 - 15.81	0.00 - 0.00
**DME+AKI**										
No, n (%)	846 (96.5)	98 (88.3)		817 (95.8)	127 (96.2)	198 (91.2)	235 (99.6)	30 (96.8)	43 (100)	1 (100)
Yes, n (%)	31 (3.5)	13 (11.7)		36 (4.2)	5 (3.8)	19 (8.8)	1 (0.4)	1 (3.2)	0	0
95% CI (%)	2.41 - 4.98	6.39 - 19.19		2.97 - 5.80	1.24 - 8.62	5.35 - 13.34	0.01 - 2.34	0.08 - 16.70	0.00 - 0.00	0.00 - 0.00

Abbreviations: AKI, acute kidney injury; ALL, acute lymphoblastic leukemia; CNS, central nervous system; DME, delayed methotrexate elimination; NHL, non-Hodgkin lymphoma; PCNSL, primary CNS lymphoma
