# Supplementary material for: High-Dose Methotrexate at All Ages: Safety, Efficacy, and Outcomes from the HDMTX European Registry
Source: Cancers (Basel). 2025 Dec 30;18(1):124. doi: 10.3390/cancers18010124 (PMC12784913; doi:10.3390/cancers18010124)
Supplement: Supplementary file 1 [file cancers-18-00124-s001.zip › Table S2.pdf]

Table S2. Incidence and characteristics of secondary endpoints by cancer type

	All courses (N=2501)	ALL (N=988)	PCNSL (N=853)	NHL (N=349)	Osteosarcoma (N=267)	Other CNSC (n=44)
**Hospital LOS for chemotherapy**, days						
Median	4.4	3.8	4.9	6.8	4.1	4.0
Q1, Q3	3.8, 6.3	3.0, 5.3	4.1, 6.1	5.0, 15.0	3.8, 4.7	4.0, 7.0
Range	0.0 - 375.2	1.6 - 375.2	0.0 – 62.0	2.8 - 86.0	0.3 - 124.1	3.2 - 119.9
Missing	488	204	139	28	109	8
**Days before subsequent cycle,** days						
Median	17.9	14.0	20.6	25.7	13.1	38.9
Q1, Q3	14.0, 27.8	14.0, 18.0	14.1, 28.0	21.0, 30.0	7.0, 32.5	14.0, 19.0
Range	5.9 - 959.7	12.0 - 387.0	10.7 - 959.7	8.8 - 722.0	5.9 – 146.9	12.0 - 70.9
N-Miss	613	292	167	112	32	10
**Delay in subsequent cycle**						
No, n (%)	1421(56.8)	547 (55.4)	515 (60.4)	171 (49.0)	164 (61.4)	24 (54.5)
Yes, n (%)	300 (12.0)	124 (12.6)	133 (15.6)	19 (5.4)	16 (6.0)	8 (18.2)
Last course in treatment cycle	613 (24.5)	292 (29.6)	167 (19.6)	112 (32.1)	32 (12.0)	10 (22.7)
Unknown	167 (6.7)	25 (2.5)	38 (4.5)	47 (13.4)	55 (20.6)	2 (4.5)
95% CI (%)	54.93 - 58.85	52.20 - 58.49	57.00 - 63.68	43.64 - 54.37	55.30 - 67.29	38.85 - 69.61
**Methotrexate dose reduction in subsequent HDMTX course**						
No, n (%)	2036 (81.4%)	893 (90.4)	541 (63.4)	312 (89.4)	246 (92.1)	44 (100)
Yes, n (%)	465 (18.6%)	95 (9.6)	312 (36.6)	37 (10.6)	21 (7.9)	0
95% CI (%)	17.09 - 20.17	7.85 - 11.63	33.34 - 39.91	7.58 - 14.32	4.93 - 11.77	0.00 - 0.00
**Methotrexate dose omission in subsequent HDMTX course**						
No, n (%)	2425 (97.0%)	971 (98.3)	823 (96.5)	325 (93.1)	263 (98.5)	43 (97.7)
Yes, n (%)	76 (3.0%)	17 (1.7)	30 (3.5)	24 (6.9)	4 (1.5)	1 (2.3)
95% CI (%)	2.40 - 3.79	1.01 - 2.74	2.39 - 4.98	4.46-10.06	0.41 - 3.79	0.06 - 12.02
**Rehospitalization for toxicity management**						
No, n (%)	2354 (94.1%)	921 (93.2%)	844 (98.9)	308 (88.3)	245 (91.8)	36 (81.8)
Yes, n (%)	147 (5.9%)	67 (6.8%)	9 (1.1)	41 (11.7)	22 (8.2)	8 (18.2)
95% CI (%)	4.99 - 6.87	5.29 - 8.53	0.48 - 1.99	8.56 - 15.60	5.24 - 12.21	8.19 - 32.71
**Hospital LOS for toxicity management**, days						
Median	4.9	3.5	15.1	7.5	3.2	7.6
Q1, Q3	2.1, 9.5	1.9, 6.2	8.3, 20.9	5.0, 13.2	1.3, 4.8	2.0, 13.8
Range	0.0 - 172.1	0.0 - 172.1	5.0 - 36.2	1.4 - 55.8	0.0 - 95.8	0.9 - 119.9
Missing	2354	921	844	308	245	36

Abbreviations: ALL, acute lymphoblastic leukemia; CNSC, central nervous system cancers; NHL, non-Hodgkin lymphoma; HDMTX, high-dose methotrexate; PCNSL, primary CNS lymphoma
