# Supplementary material for: High-Dose Methotrexate at All Ages: Safety, Efficacy, and Outcomes from the HDMTX European Registry
Source: Cancers (Basel). 2025 Dec 30;18(1):124. doi: 10.3390/cancers18010124 (PMC12784913; doi:10.3390/cancers18010124)
Supplement: Supplementary file 1 [file cancers-18-00124-s001.zip › Table S3.pdf]

Table S3. Three- and 5-year event-free survival and overall survival estimates by cancer type

	*EFS (%)				*OS (%)			
	3-year	95% CI	5-year	95% CI	3-year	95% CI	5-year	95% CI
ALL	88.1	0.841-0.922	84.0	0.787-0.896	93.6	0.907-0.966	91.9	0.881-0.958
PCNSL	47.2	0.392-0.567	37.3	0.286-0.488	77.9	0.710-0.853	71.8	0.631-0.818
NHL	66.1	0.574-0.761	66.1	0.574-0.761	75.8	0.678-0.848	72.4	0.636-0.824
Osteosarcoma	44.8	0.299-0.671	41.4	0.268-0.638	82.8	0.701-0.977	60.7	0.450-0.820
Other CNSC	48.0	0.246-0.938	48.0	0.246-0.938	70.0	0.467-1.000	70.0	0.467-1.000
Log rank	$p < 0.001$				$p < 0.001$			

* Survival estimates from start of the first HDMTX course administered
