# Supplementary material for: High-Dose Methotrexate at All Ages: Safety, Efficacy, and Outcomes from the HDMTX European Registry
Source: Cancers (Basel). 2025 Dec 30;18(1):124. doi: 10.3390/cancers18010124 (PMC12784913; doi:10.3390/cancers18010124)
Supplement: Supplementary file 1 [file cancers-18-00124-s001.zip › Table S4.pdf]

Table S4. Event-free and overall survival estimates by occurrence of the primary endpoints

Primary Endpoint	*EFS (%)				*OS (%)			
	3-year	95% CI	5-year	95% CI	3-year	95% CI	5-year	95% CI
**AKI**								
No	70.7	0.656-0.662	67.5	0.618-0.737	84.1	0.800-0.884	79.5	0.743-0.851
Yes	71.5	0.661-0.774	65.9	0.595-0.729	86.9	0.827-0.912	81.5	0.762-0.873
Log rank	p = 0.496				p = 0.276			
**DME**								
No	75.3	0.709-0.798	71.3	0.664-0.766	88.9	0.858-0.922	82.5	0.779-0.873
Yes	61.6	0.547-0.695	55.9	0.478-0.653	77.4	0.712-0.841	76.0	0.695-0.832
Log rank	p < 0.001				p = 0.003			
**DME + AKI**								
No	72.6	0.687-0.769	68.2	0.735-0.732	85.8	0.827-0.891	80.1	0.760-0.845
Yes	61.6	0.516-0.735	57.0	0.463-0.701	82.2	0.738-0.916	82.2	0.738-0.916
Log rank	p = 0.095				p = 0.714			

* Survival estimates from start of the first HDMTX course administered
