# Supplementary material for: High-Dose Methotrexate at All Ages: Safety, Efficacy, and Outcomes from the HDMTX European Registry
Source: Cancers (Basel). 2025 Dec 30;18(1):124. doi: 10.3390/cancers18010124 (PMC12784913; doi:10.3390/cancers18010124)
Supplement: Supplementary file 1 [file cancers-18-00124-s001.zip › Table S5.pdf]

Table S5. Total number of courses included in the Registry by investigational site (N=3165)

**Investigational Site (City, Country)**	**Number of courses registered**
La Timone Hospital, Marseille, France	659
Hospital Universitario Reina Sofia, Cordoba, Spain	364
Medizinische Hochschule Hannover, Hannover, Germay	340
IRCCS San Raffaele Scientific Institute	325
Vall d'Hebron Barcelona Hospital (VHIR), Barcelona, Spain	321
University of Milano-Biococca	304
Nottingham University Hospital, Nottingham, United Kingdom	304
Vall d'Hebron Barcelona Hospital (VHIO), Barcelona, Spain	300
Institut Universitaire du Cancer Toulouse Oncopole, Toulouse, France	85
University College London Hospitals, London, United Kingdom	79
University of Freiburg, Freiburg, Germany	59
Charité-Univeristatsmedizin Berlin, Berlin, Germany	25
TOTAL	3165
