# Supplementary figures and images for: High-Dose Methotrexate at All Ages: Safety, Efficacy, and Outcomes from the HDMTX European Registry
Source: Cancers (Basel). 2025 Dec 30;18(1):124. doi: 10.3390/cancers18010124 (PMC12784913; doi:10.3390/cancers18010124)

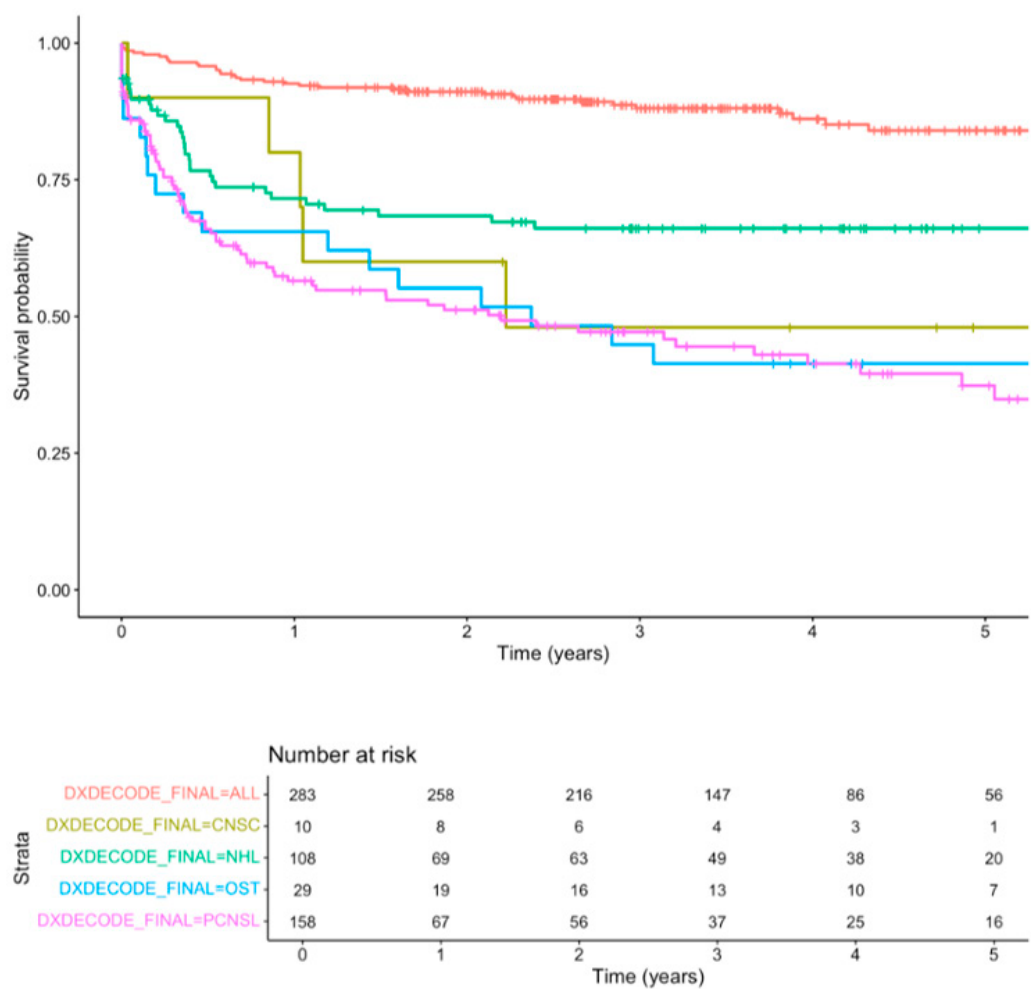

Figure S1. Kaplan-Meier estimates for EFS by cancer type from start of HDMTX administration

Supplement: Supplementary file 1 [file cancers-18-00124-s001.zip › Figure S1.pdf]

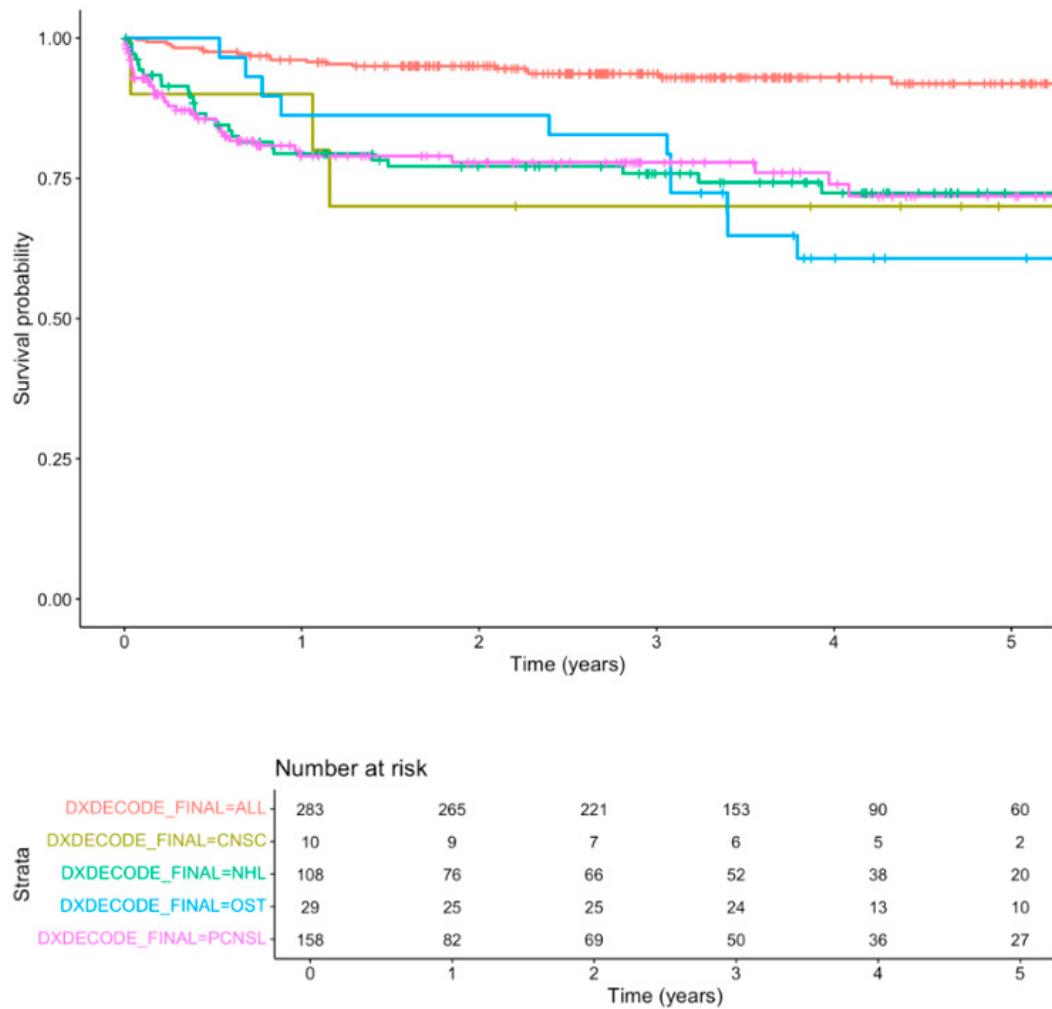

Figure S2. Kaplan-Meier estimates for OS by cancer type from start of HDMTX administration

Supplement: Supplementary file 1 [file cancers-18-00124-s001.zip › Figure S2.pdf]
